# Normative data for certain vocal fold biomarkers among young normophonic adults using ultrasonography

**DOI:** 10.1007/s00405-023-08025-6

**Published:** 2023-05-23

**Authors:** Santosh Rai, Divya Ramdas, Nidhi Lalu Jacob, Gagan Bajaj, Radish Kumar Balasubramanium, Jayashree S. Bhat

**Affiliations:** 1 Department of Radiodiagnosis and Imaging, Kasturba Medical College, Mangalore, Manipal Academy of Higher Education, Manipal, 575001 Karnataka India; 2 Department of Audiology and Speech Language Pathology, Kasturba Medical College, Mangalore, Manipal Academy of Higher Education, Manipal, 575001 Karnataka India; 3 Department of Audiology and Speech Language Pathology, Nitte Institute of Speech and Hearing, Deralakatte, Mangalore, Karnataka India

**Keywords:** Laryngeal ultrasonography, Vocal fold length, Vocal fold displacement velocity, Young adults

## Abstract

**Purpose:**

The current study aimed to profile vocal fold morphology, vocal fold symmetry, gender and task-specific data for vocal fold length (VFL) and vocal fold displacement velocity (VFDV) in young normophonic adults in the age range of 18–30 years using ultrasonography (USG).

**Methods:**

Participants underwent USG across quiet breathing, /*a*/ phonation and /*i*/ phonation tasks, and acoustic analysis was conducted to explore the relationship between USG and acoustic measures.

**Results:**

The study found that males have longer vocal folds than females, and overall greater velocities were observed in /*a*/ phonation, followed by /*i*/ phonation, with the lowest velocity observed in the quiet breathing task.

**Conclusions:**

The obtained norms can be used as a quantitative benchmark for analyzing the vocal fold behavior in young adults.

## Introduction

Ultrasonography (USG) has become increasingly popular for evaluating laryngeal functions, offering health professionals, like laryngologists and speech-language pathologists (SLPs), a simple and non-invasive diagnostic tool [1]. While other imaging techniques, such videokymography, stroboscopy, and high-speed digital imaging, directly visualize the vocal folds, they have limitations such as heavy imaging data and higher costs [2]. USG offers a convenient and accessible alternative [3]. Its non-invasive nature promotes safe usability and better patient tolerance [4]. Recent studies have shown that USG could be used as a reliable method for assessing vocal folds [5] and offer better methodological homogeneity for evaluating laryngeal functions [6].

The use of USG enables the examination of anatomical and physiological characteristics of the laryngeal system, including mucosal wave measurement, muscle length, and speech valve adaption [7]. Studies have shown that USG has a high visualization rate of the thyroid and cricoid structures making it a viable supplementary and alternative technique for laryngeal evaluation [8]. USG has been found to be a reliable, non-invasive, and affordable tool for assessing vocal cords with high sensitivity and specificity in diagnosing vocal cord palsy [9]. Vocal fold mobility can be evaluated preoperatively by measuring the inter arytenoid distance or through recorded video observations [10]. USG has also been found to have a high sensitivity for detecting recurrent laryngeal nerve palsy in post-thyroidectomy patients [11]. It has been used as a reference for measuring the area and angles of the vocal folds in healthy adults [12].

Besides informing about the mobility of the vocal cords, USG has the potential to offer very specific parameters like vocal fold displacement velocity (VFDV) and vocal fold lengths (VFL). The VFDV represents a component of mucosal wave velocity. This measurement has been proposed as an alternative to assess vocal fold stiffness, which is essential for the generation of mucosal waves [13–15]. The VFL, which is used to study vocal fold behavior [16], is typically calculated by measuring the length of the vocal fold from the anterior commissure of the arytenoid cartilage to the edge of the vocal process [17]. Although ultrasonography (USG) has demonstrated great effectiveness in clinical practice for laryngeal examination, there are currently no established norms for laryngeal examination. Studies on measures such as VFL and VFDV have been conducted using various other instruments, such as direct laryngoscopy and high-speed video endoscopy, with only a few investigations considering the usefulness of USG for such measurements [18, 19].

While USG is a low-cost, non-invasive, non-radioactive, and safe method for clinical evaluation [20, 21], more research is needed to establish its standard use by concerned health professionals in comparison to other diagnostic techniques. The use of USG for determining the appropriate treatment for laryngeal issues has not been thoroughly studied and studies have called for more scientific evidence before USG can be used in a clinical setting [6]. The lack of adequate normative evidence has hindered the widespread adoption of USG as a means of evaluating patients [22].

To facilitate the translation of USG into clinical practice, for laryngeal functions, and establish consensus on the priority areas in this regard, a recent study implemented the nominal group technique to develop consensus among an international USG working group over two formal meetings [1]. The study provided a framework wherein several aspects pertaining to stakeholders, training and protocols, and evidence base and metrics were highlighted. Establishing normative data for the potential biomarkers of the vocal folds, using USG, among individuals of different age groups under diverse conditions emerged as a key priority area, besides reliability and validity. The study further emphasized that although some initial data are available regarding the thickness and echo intensity of submental and tongue muscles as well as hyoid bone movement in healthy young and aging adults, there are no established standards for assessing vocal fold biomarkers. In order for USG to be a diagnostic tool rather than just a screening or biofeedback tool, it must be able to differentiate between significant changes. Discriminant validity is critical for determining the limits of reliability and depends on having normative data for comparison.

In view of the advantages offered by USG, its scope and potential to evaluate the biomarkers of the vocal folds, and the recommendations made by the international USG working group to establish age and condition-specific normative data for these biomarkers, the present study aimed at establishing normative data for certain anatomical and physiological aspects of vocal folds in young normophonic adults using USG. The specific objectives of the study were to visualize the vocal fold morphology and symmetry, and establish normative data for VFL and VFDV among young normophonic adults using USG. Since these parameters are may have a relation to the acoustic correlates, the study also explored the acoustic correspondence of the vocal fold parameters obtained through USG with the parameters like fundamental frequency, frequency range, jitter, and cepstral peak prominence.

## Methods

The study followed a cross-sectional research design and was approved by the institutional ethical committee (IEC-KMC-MLR03-2021/83). Informed consent was obtained from all the participants prior to their enrollment into the study.

### Participants

54 normophonic individuals within the age range of 18–30 years(27 males and 27 females; mean age ± S.D:21.5 ± 2.24 years) were recruited for the study using the purposive sampling method based on the sample size calculation formula on the reference study [17], [*n* = (*Z*^2^ *σ*^2^/*d*^2^), where, *Z* = 1.96 (with 95% confidence level), *σ* = 1.337, *d* = 0.4].

The study included participants with normal phonatory functions and a “normal” grade on the GRBAS scale [23] while excluding those with self-reported smoking or vocal abuse, vocal tract irregularities, vocal fold pathologies, self-reported history of dysphonia or upper respiratory disorders, pathologies around the neck, reflux conditions, dense thyroid cartilages, or unsatisfactory visualization during USG. Based on these criteria, four participants were excluded, leaving data collected from 50 participants in the study.

### Procedure

A comprehensive case history form was used to obtain information on the devised selection criteria and perceptual voice screening was done using GRBAS scale [23]. The study used 3D/4D GE LogiqV5 transcutaneous B-mode ultrasound, performed by a qualified radiologist with over 15 years of experience. The participants were positioned supine with their necks extended for better visualization of the laryngeal structures, and USG was performed using a multi-frequency linear array transducer (5–12 MHz) with the probe placed horizontally on the lateral aspect of the thyroid lamina. The Doppler gate was placed on the vocal folds in an oblique lateral position. The line shift and scale on the USG were adjusted until the maximum amplitude was obtained. The length of the vocal folds between the anterior commissure and arytenoid cartilage was measured during quiet breathing and vowel phonation tasks (/*a*/ and /*i*/). The USG parameters, VFL, and VFDV, were recorded, and the data were noted on an observation sheet during the procedure. VFL and VFDV were measured using the color mode with a pulse repetition rate of 10,000 Hz and a frame rate of 7 Hz. The measured velocities ranged from 0 to 100 cm/s. Participants with a limited acoustic window to visualize structures such as arytenoids, thyroid lamina, and true and false vocal folds were excluded.

**Fig. 1:**
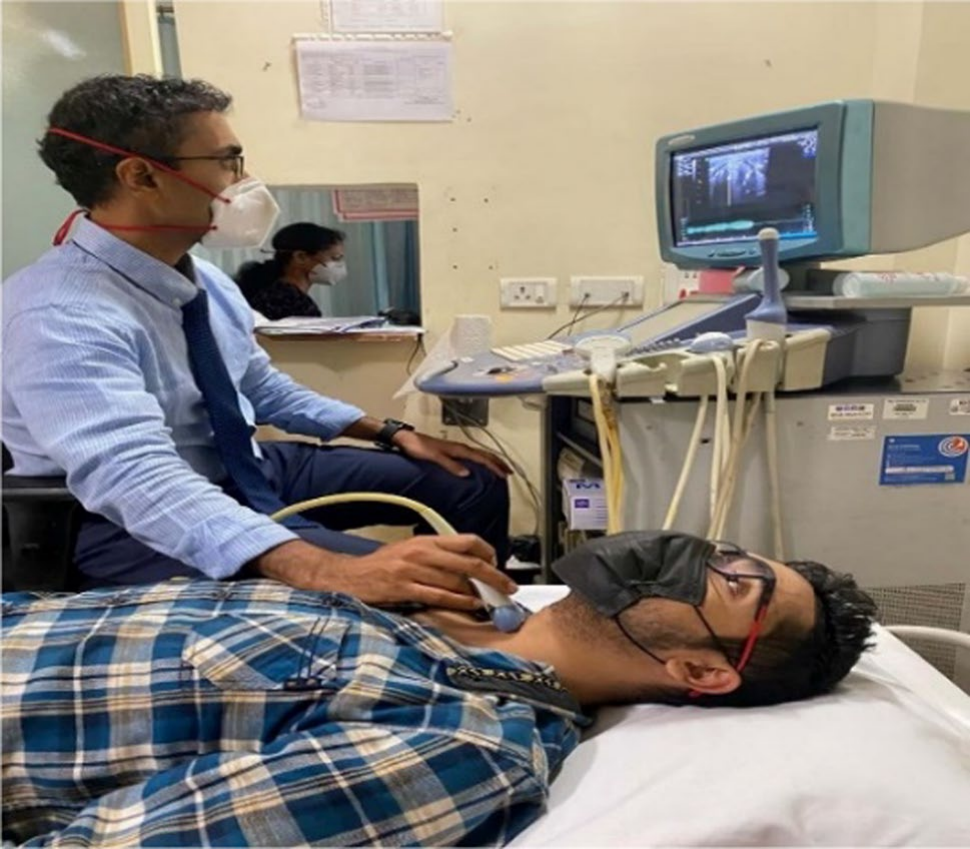
USG recording

**Fig. 2:**
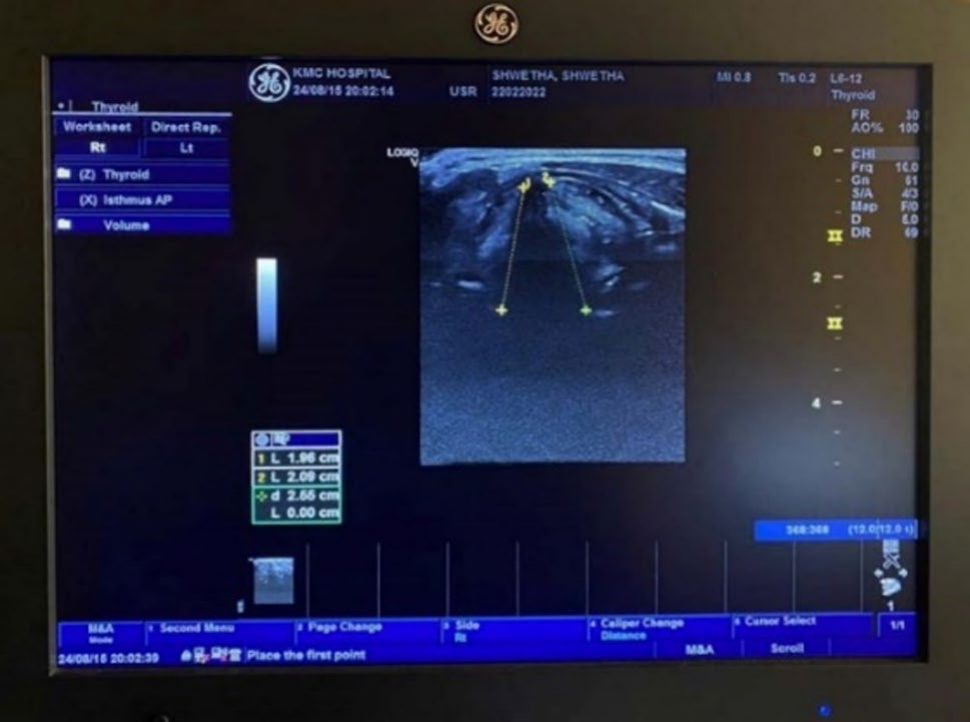
Vocal fold length measurement

**Fig. 3:**
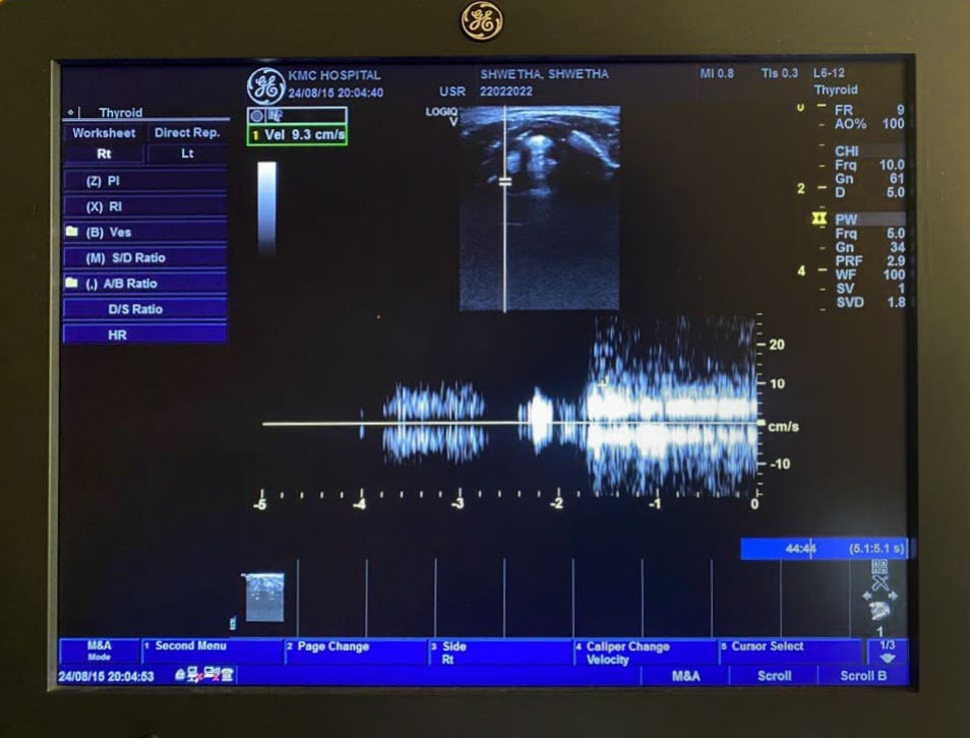
Vocal fold displacement velocity measurement

Illustrations depicting USG recording and measurement of VFL and VFDV have been shown in Figs. 1, 2 and 3.

Acoustic analysis was performed using Praat software (Version 6.2.03) installed on a Lenovo laptop (Model:81WEIdeapad315IIL05), and the samples were recorded using a Jabra Evolve (40 UC) headphone with an inbuilt microphone. The recordings were made in a noise-free environment, with the participants sitting upright and relaxed, and the microphone positioned 5 cm away from the mouth at a 45° angle. Participants were instructed to phonate vowels (/*a*/, /*i*/) and sustain the phonation for 3–5 s, and three trials were recorded for each. The best sample [24] was selected for the analysis of the acoustic measures, including fundamental frequency (f0), jitter, and cepstral peak prominence (CPP). Additionally, the participants performed a pitch glide task on /*a*/, which was analyzed for the frequency range.

### Data analysis

The study assessed vocal fold morphology and symmetry using a rubric that categorized vocal fold morphology into adequately visualized, poorly visualized, or not visualized, and vocal fold symmetry into Grades I to III based on mobility and position [25]. The data obtained were analyzed using frequency measures.

Quantitative data were subjected to statistical analysis using SPSS software (Version:16.0). The mean and standard deviation of the USG measurements, VFL, and VFDV were subjected to descriptive statistics. Mixed model ANOVA was used to determine the gender-specific difference of USG parameters across the tasks. Pearson’s correlation was used to study the relationship between VFL and VFDV with the acoustic measures.

## Results

The present study aimed to profile vocal fold morphology, vocal fold symmetry, gender and task-specific VFL, VFDV using USG. Furthermore, the study explored the relationship between VFL and VFDV with acoustic measures such as f0, frequency range, jitter, and CPP in young normophonic adults.

### Vocal fold morphology and symmetry

Depending on the visibility of the laryngeal structures, such as the cricoid cartilage, thyroid cartilage, false vocal folds, true vocal folds, anterior commissure of vibrating vocal folds, and posterior commissure of vibrating vocal folds, the anatomical state of vocal structures of all participants (100%) was labeled ‘adequately visualized’. With respect to vocal fold symmetry, depending on the pattern of vocal fold movements, the physiological state of vocal folds of all the participants (100%) was labeled as ‘Grade I (Normal mobility)’.

### Vocal fold length

**Fig. 4:**
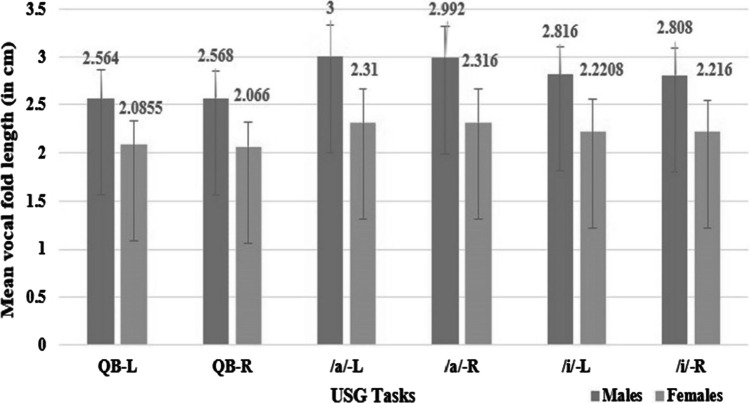
Descriptive statistics for vocal fold length of the right (R) and left (L) vocal fold across quiet breathing (QB), /*a*/ phonation, /*i*/ phonation in males and females. *QB* quiet breathing, */a/* /*a*/phonation, /*i*/ /i/phonation, *L* left vocal fold, *R* right vocal fold

The mean VFL during quiet breathing was measured to be 2.08 cm (SD-0.24 cm) and 2.06 cm (SD-0.25 cm) in females and 2.56 cm (SD-0.30 cm) and 2.56 cm (SD-0.28 cm) among males for left and right vocal fold, respectively. During /*a*/ phonation, the VFL in females was 2.31 cm (SD-0.35 cm) and 2.31 cm (SD-0.34 cm); and 3 cm (SD-0.32 cm) and 2.99 cm (SD-0.32 cm) among males for left and right vocal fold, respectively. During /*i*/ phonation, the length of the vocal folds was 2.22 cm (SD-0.33 cm) and 2.21 cm (SD-0.33 cm) among females; and 2.81 cm (SD-0.28 cm) and 2.8 cm (SD-0.28 cm) among males for the left and right vocal folds, respectively (Fig. 4).

The results of the mixed model ANOVA revealed that the vocal folds were significantly longer during /*a*/ phonation, followed by /*i*/ phonation, and quiet breathing for the left [*F* (2, 46) = 53.24, *p* = 0] and right [*F* (2, 47) = 57.20, *p* = 0] vocal folds. Gender-specific analysis revealed that the VFL was significantly longer in males than in females in all tasks for the left[*F* (2,46) = 5.07, *p* = 0.01] and right [*F* (2,47) = 4.10, *p* = 0.023] vocal folds.

### Vocal fold displacement velocity

**Fig. 5:**
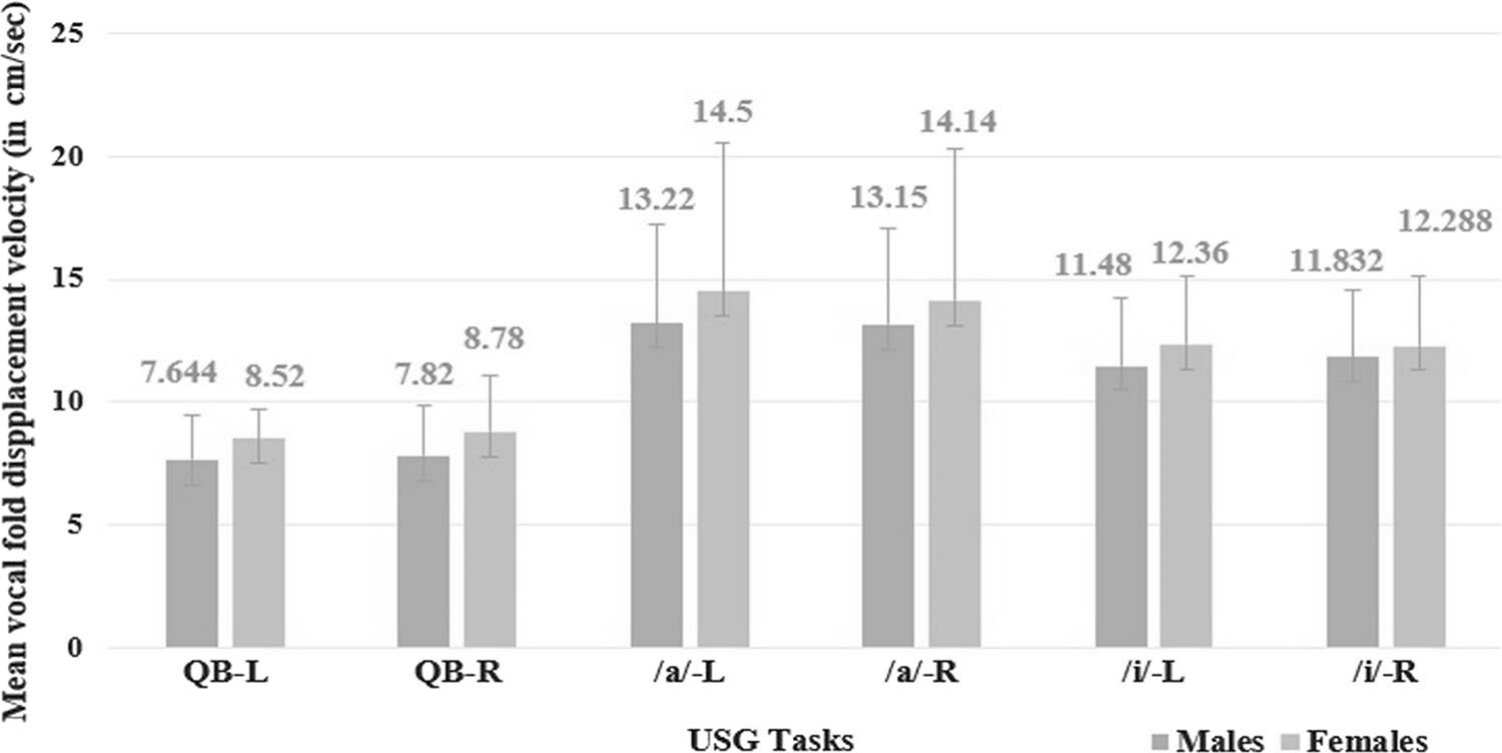
Descriptive statistics for vocal fold displacement velocity of the right (*R*) and left (*L*) vocal folds across quiet breathing (QB), /*a*/ phonation, /*i*/ phonation in males and females. *QB* quiet breathing, */a/* /*a*/phonation, */i/* /*i*/phonation, *L* left vocal fold, *R* right vocal fold

The mean VFDV during quiet breathing was measured to be 8.52 cm/s (SD-1.15 cm/s) and 8.78 cm/s (SD-2.30 cm/s) in women and 7.64 cm/s (SD-1.79 cm/s) and 7.82 cm/s (SD-2.05 cm/s) in men for the left and right vocal fold, respectively. During /*a*/ phonation, VFDV in females was 14.5 cm/s (SD-6.09 cm/s) and 14.14 cm/s (SD-6.19 cm/s); and 13.22 cm/s (SD-4.0 cm/s) and 13.15 cm/s (SD-3.93 cm/s) among males for left and right vocal fold, respectively. During /*i*/ phonation, the velocity of the vocal folds was 12.36 cm/s (SD-2.75 cm/s) and 12.28 cm/s (SD-2.88 cm/s) among females; and 11.48 cm/s (SD-2.74 cm/s) and 11.83 cm/s (SD-2.72 cm/s) among males for left and right vocal fold, respectively (Fig. 5).

The results revealed that the VFDV was highest during /*a*/ phonation followed by /*i*/ phonation and least during quiet breathing; for left [(*F* (2,47) = 59.11, *p* = 0] and right [*F* (2,47) = 38.64, *p* = 0] vocal folds. However, the difference between tasks was not statistically significant with respect to gender for the left [*F* (2,47) = 0.09, *p* = 0.912] and right [*F* (2,47) = 0.48, *p* = 0.622] vocal folds.

### Correlation between USG parameters and acoustic measures

Pearson’s correlation was performed to derive the relationship between the USG parameters obtained (VFL and VFDV) and acoustic measures (f0, frequency range, jitter, and CPP).

For /*a*/ phonation, a statistically significant moderate inverse correlation was obtained between VFL and f0 (*L*, *r* (50) = − 0.639, *p* =  < 0.001), (*R*, *r* (50) = − 0.636, *p* =  < 0.001). No statistically significant correlations were observed between VFL, jitter, and CPP. Furthermore, a statistically significant weak inverse correlation was obtained between the frequency range derived during pitch glide /*a*/ and the VFL, indicating that, as the VFL increased, the frequency range decreased (*L*, *r* (50) = − 0.386, *p* = 0.006), (*R*, *r* (50) = − 0.378, *p* = 0.007) (Table 1).

**Table 1:** Correlation between vocal fold length and acoustic measures (fundamental frequency (f_0_), frequency range, jitter and cepstral peak prominence)

Parameters	Fundamental frequency (f_0_) (r,p)	Frequency range (r,p)	Jitter (r,p)	CP P (r,p)
VFL /a/- L	− 0.639*** ** < 0.001**	− 0.386** **0.006**	0.014 0.925	0.352 0.012
VFL /a/- R	− 0.636*** ** < 0.001**	− 0.378** **0.007**	0.014 0.921	0.337 0.017
VFL /i/- L	− 0.600*** ** < 0.001**		0.163 0.259	0.321 0.023
VFL /i/- R	0.597*** ** < 0.001**		0.160 0.268	0.320 0.023

*VFL* vocal fold length, */a/* /*a*/phonation, */i/* /*i*/phonation, *L* left vocal fold, *R* right vocal fold, *CPP* cepstral peak prominence

For /*i*/ phonation, a statistically significant moderate inverse correlation was obtained between VFL and f0 (*L*, *r* (50) = − 0.600, *p* < 0.001) and (*R*, *r* (50) = 0.597, *p* < 0.001), indicating that as VFL increased, f0 decreased (Table 1). No statistically significant correlation was observed between VFL, jitter, and CPP.

With respect to VFDV, no statistically significant correlation was obtained with f_0_, frequency range, jitter and CPP (Table 2).

**Table 2:** Correlation between vocal fold displacement velocity and acoustic measures (fundamental frequency (f_0_), frequency range, jitter and cepstral peak prominence)

Parameters	Fundamental frequency (f_0_) (r,p)	Frequency range (r,p)	Jitter (r,p)	CPP (r,p)
VFDV /a/ L	0.129 0.371	0.015 0.917	**− **0.097 0.502	**− **0.006 0.966
VFDV /a/ R	0.102 0.481	0.002 0.989	**− **0.088 0.543	0.032 0.827
VFDV /i/ L	0.196 0.173		**− **0.168 0.244	0.075 0.604
VFDV /i/ R	0.129 0.373		**− **0.075 0.605	0.117 0.418

*VFDV* vocal fold displacement velocity, */a/* /*a*/phonation, */i/* /*i*/phonation, *L* left vocal fold, *R* right vocal fold, *CPP* cepstral peak prominence

## Discussion

Recently, USG has become popular owing to its capability to identify typical and atypical laryngeal dynamics with several advantages over traditional measures, such as low cost, safety, speed, painlessness, accessibility, good tolerance, non-invasiveness, and precision [3, 4]. Establishing USG-based normative values for laryngeal structures is essential for healthcare professionals for diagnostic and therapeutic purposes [1]. In this study, vocal fold morphology, symmetry, task-specific, and gender-specific norms for VFL and VFDV were analyzed using USG. Additionally, the relationship between USG parameters and acoustic measures, such as f0, frequency range, jitter, and CPP, were examined. Vocal fold morphology was evaluated in healthy participants based on landmarks such as the thyroid cartilage, arytenoid cartilage, false vocal folds, true vocal folds, cricoid cartilage, and the anterior, and posterior commissure of the vocal folds. The landmarks were well visualized in all healthy individuals, which is consistent with previous reports on the examination of vocal structures in normophonic adults [26, 27].

The study evaluated vocal fold symmetry based on vocal fold mobility, and all participants demonstrated perfect symmetry (Grade I). A previous study reported similar symmetrical movements of the vocal folds before and after thyroid surgery. Patients were classified as ‘grade I’ before surgery, showing normal symmetry, and grades II and III after surgery, indicating decreased or no vocal fold movements [25].

The study aimed to establish norms for VFL and VFDV during active (phonation of /*a*/, /*i*/) and passive maneuvers (quiet breathing) to aid in detecting laryngeal diseases. VFL and VFDV measurements during active and passive maneuvers are proved effective in detecting specific laryngeal diseases [28]. Conventional techniques for measuring VFL accurately may be limited by internal structural movement, but USG can accurately measure VFL even during motion-based tasks such as phonation and singing [29]. Previous studies have measured VFL in healthy adults, with the results being 13.8 mm ± 2.92 mm in males and 10.7 mm ± 1.63 mm in females [30], 22.09 mm ± 3.07 mm in males, and 17.55 mm ± 0.92 mm in female [31], 15.3 mm and 13.5 mm in males and females [32], 16.11 mm ± 2.62 mm in males and 14.10 mm ± 1.54 mm in females [33] and 24.9 mm and 17.5 mm in males and females, respectively [34]. Males generally have longer vocal folds than females. This could be due to anatomical differences and the influx of testosterone during puberty, resulting in larger vocal fold muscles, increased mass, and prominent thyroid structures in males [35]. The norms for the VFL obtained in this study are consistent with previous findings. Furthermore, VFL was shortest during quiet breathing, likely because there are minimal changes in the vocal folds during this task due to limited tilting movement of the cricoid on the thyroid [29]. Additionally, the VFL was longer during the phonation of /*a*/ compared to /*i*/, possibly because the narrower opening of the glottis during /*i*/ resulted in a shorter VFL [36].

VFDV is an indicator of the physiological condition and stiffness of the vocal fold cover. Studies have shown that VFDV ranges from 58 to 68 cm/s in young healthy individuals and between 65 and 140 cm/s in individuals with vocal fold pathologies before thyroidectomy, but is less than 30 cm/s post-surgery [37]. Laser Doppler vibrometry indicated an average velocity of 45 mm/s during phonation [38]. Patients undergoing thyroid surgery were found to exhibit VFDV at speeds ranging from 80 to 150 cm/s [5]. However, the present study obtained lower VFDV values, which may be due to the lateral displacement of the USG probe. Medial placement of the USG probe can result in poor image quality due to strong reverberation artifacts and anisotropic reflection of the ultrasound at the medial air-mucosa interface of the vocal folds [39, 40]. To avoid this, the gate was not centrally positioned. In addition, placing the gate at the center would require a change in the scale and baseline of the graph, which is another limitation. A study using a similar lateral placement of the USG probe found VFDV values ranging from 5 to 16 cm/s for quiet breathing and 9–110 cm/s for phonation in healthy adults [18]. The velocity of vocal fold movement tends to increase with an increase in the intensity of target stimuli and decrease with increasing pitch [18]. When producing the vowel sound /*a*/, the vocal tract is more open, which causes a higher vocal fold vibratory displacement (VFDV), resulting in higher intensities compared to the vowel sound /*i*/. This finding is consistent with the existing literature, although other factors such as the length of the vocal tract and intrinsic vowel characteristics may also have contributed. The present study observed an increase in VFDV during speaking and phonatory tasks compared with quiet breathing.

The correlation between VFL and f0 may be supported by previous studies, where changes in pitch have been discussed with respect to morphological changes in the vocal folds. The f0 of vocal folds is a product of the nonlinear behaviors of stiffness and tension, which further shortens or elongates the vocal folds, thereby changing the pitch of the voice [41]. The frequency of the vibratory characteristics of vocal folds is most often associated with length, mass, and tension, such that an increase in f0 is associated with increasing tension, decreasing length, and decreasing mass [41]. f0 modulations are achieved by changes in the VFL [42].

Developing a standardized set of normative data for USG is crucial to ensure accurate distinction between normal and abnormal laryngeal function [1]. Establishing the discriminant validity of ultrasound imaging requires normative data [1], and the current study’s findings aids in this regard.

## Conclusion

USG is an effective, non-invasive, and affordable tool for assessing laryngeal functions. USG provides specific parameters like VFDV and VFL which are useful in diagnosing voice disorders. However, the lack of available normative data limits its use in clinical practice. This study aimed to profile vocal fold behavior in young adults using USG, allowing for precise diagnosis and treatment planning. The obtained norms can be used as a quantitative benchmark for analyzing vocal fold behavior in young adults. Overall, USG can serve as an effective tool for SLPs to evaluate and diagnose voice disorders.

### Clinical implications

The normative database obtained for VFL and VFDV using USG can serve as a quantitative reference for identifying normal vocal fold function in young normophonic adults, aiding in the diagnosis of voice disorders. The data can also be used as a baseline for monitoring vocal fold status during voice intervention, leading to better patient outcomes and more individualized treatments. The study's findings provide a crucial step towards increasing the utilization of USG by healthcare professionals for diagnosing and treating voice disorders, which was previously limited due to a lack of normative database.

### Limitations and future directions

Future studies should expand on the current study’s normative database for USG by including additional parameters like glottal area and interarytenoid distance. To achieve better population representation, larger sample sizes will be necessary. Monitoring vocal loudness during USG evaluations could provide better insights, and normative data for other age groups are needed to explore age-related trends. Developing a comprehensive USG training protocol would limit operator differences in interpreting the results obtained using this technique.

## Data Availability

The datasets generated during and/or analysed during the current study are available from the corresponding author on request.
