# Correction: Normative data for certain vocal fold biomarkers among young normophonic adults using ultrasonography

**DOI:** 10.1007/s00405-023-08060-3

**Published:** 2023-06-12

**Authors:** Santosh Rai, Divya Ramdas, Nidhi Lalu Jacob, Gagan Bajaj, Radish Kumar Balasubramanium, Jayashree S. Bhat

**Affiliations:** 1 Department of Radiodiagnosis and Imaging, Kasturba Medical College, Mangalore, Manipal Academy of Higher Education, Manipal, 575001 Karnataka India; 2 Department of Audiology and Speech Language Pathology, Kasturba Medical College, Mangalore, Manipal Academy of Higher Education, Manipal, 575001 Karnataka India; 3 Department of Audiology and Speech Language Pathology, Nitte Institute of Speech and Hearing, Deralakatte, Mangalore, Karnataka India

**Correction: European Archives of Oto-Rhino-Laryngology** 10.1007/s00405-023-08025-6

In the original publication of the article, the following author’s name “Radish Kumar Balasubramanium” was published incorrectly as “Radish Balasubramanium”. The name is corrected in this article.

The original article was updated.

